# Longitudinal analysis of antibody responses to trachoma antigens before and after mass drug administration

**DOI:** 10.1186/1471-2334-14-216

**Published:** 2014-04-22

**Authors:** Erica Brook Goodhew, Sheri Maria G Morgan, Andrew J Switzer, Beatriz Munoz, Laura Dize, Charlotte Gaydos, Harran Mkocha, Sheila K West, Ryan E Wiegand, Patrick J Lammie, Diana L Martin

**Affiliations:** 1 Division of Parasitic Diseases and Malaria, Centers for Disease Control and Prevention, Atlanta, GA, USA; 2 The Ohio State University College of Optometry, Columbus, OH, USA; 3 St. Olaf College, Northfield, MN, USA; 4 Dana Center for Preventative Ophthalmology, Wilmer Eye Institute, Johns Hopkins University, Baltimore, MD, USA; 5 Center for Infectious Diseases, Johns Hopkins University, Baltimore, MD, USA; 6 Kongwa Trachoma Project, Kongwa, United Republic of Tanzania

## Abstract

**Background:**

Blinding trachoma, caused by the bacteria *Chlamydia trachomatis*, is a neglected tropical disease targeted for elimination by 2020. A major component of the elimination strategy is mass drug administration (MDA) with azithromycin. Currently, program decisions are made based on clinical signs of ocular infection, but we have been investigating the use of antibody responses for post-MDA surveillance. In a previous study, IgG responses were detected in children lacking clinical evidence of trachoma, suggesting that IgG responses represented historical infection. To explore the utility of serology for program evaluation, we compared IgG and IgA responses to trachoma antigens and examined changes in IgG and IgA post-drug treatment.

**Methods:**

Dried blood spots and ocular swabs were collected with parental consent from 264 1–6 year olds in a single village of Kongwa District, central Tanzania. Each child also received an ocular exam for detection of clinical signs of trachoma. MDA was given, and six months later an additional blood spot was taken from these same children. Ocular swabs were analyzed for *C. trachomatis* DNA and antibody responses for IgA and total IgG were measured in dried bloods spots.

**Results:**

Baseline antibody responses showed an increase in antibody levels with age. By age 6, the percentage positive for IgG (96.0%) was much higher than for IgA (74.2%). Antibody responses to trachoma antigens declined significantly six months after drug treatment for most age groups. The percentage decrease in IgA response was much greater than for IgG. However, no instances of seroreversion were observed.

**Conclusions:**

Data presented here suggest that focusing on concordant antibody responses in children will provide the best serological surveillance strategy for evaluation of trachoma control programs.

## Background

Trachoma is a neglected tropical disease caused by the bacterium *Chlamydia trachomatis* that is associated with ocular pathology. Trachoma causes an estimated 3.8 million cases of blindness and 5.3 million cases of low vision [1] in Africa and Southeast Asia. Active infection, defined as trachomatous inflammation–follicular (TF) or trachomatous inflammation–intense (TI), is self-limiting, but repeated infections can lead to pathology in the form of scarring (TS); trichiasis (TT), distinguishable by turned-in eyelashes rubbing against the globe of the eye; and irreversible blindness caused by corneal opacity (CO). Global efforts to eliminate blinding trachoma as a public health problem by the year 2020 are based on the components of the SAFE strategy: surgery for treatment of trichiasis, mass drug administration of antibiotics, and promotion of facial cleanliness and environmental improvements.

As elimination efforts proceed, defining programmatic endpoints becomes a priority. We have recently begun examining the utility of serological tests for monitoring and evaluation of trachoma elimination programs [2]. Antibody responses to the chlamydial antigens pgp3 and CT694 show high sensitivity and specificity for trachoma infection [2]. These studies also showed that a large percentage of children have detectable antibody responses, but exhibit no clinical signs and lack bacterial DNA in the conjunctiva, suggesting that these responses are indicators of historical rather than active infection [2]. Exactly how antibody responses can be used within a programmatic context is still under evaluation. An ideal test would be able to uniquely detect recent infections and could be integrated with serological tests for monitoring other neglected tropical disease programs or national surveillance programs [3-5]. The studies described herein sought to define parameters of anti-chlamydial antibody responses in a trachoma-endemic area and how these responses could be utilized in the framework of monitoring and evaluating trachoma control programs. To this end we (1) examined IgA responses to the previously defined antigens pgp3 and CT694 with the Luminex multiplex bead assay as possible indicators of recent infection and (2) monitored IgG and IgA responses in children at baseline and 6 months after MDA with azithromycin to determine whether seroreversion occurs after treatment.

## Methods

### Study population

Studies were conducted in the Kongwa District of Tanzania as part of ongoing clinical trials to evaluate the impact of alternative models of community-wide treatment with azithromycin and to compare nucleic acid amplification test methods [6,7]. Children 1–6 years of age were recruited from a single community to maximize the number of PCR-positive samples within the study. After baseline data collection, all participants (N = 264) received a single oral dose of azithromycin as part of a mass drug administration (MDA) program. At baseline, clinical examinations were performed, and dried blood spots (DBS) and conjunctival eye swabs for PCR were collected. At 6 months after MDA, only dried blood spots were collected. A total of 173 children were examined at both baseline and the six month post-treatment period, and analysis was limited to this subset of children. Parents or guardians provided written informed consent for children participating in the study. The study was approved by The Institutional Review Board of the Johns Hopkins University School of Medicine (Baltimore, MD) and the Tanzanian National Institute for Medical Research. Researchers at Centers for Disease Control and Prevention (Atlanta, GA) received de-identified samples and were non-engaged.

#### Grading of ocular Trachoma

Clinical exams, using an expansion of the WHO simplified grading scheme [8,9] were performed on 264 children aged 1 to 6 years who were randomly selected from a single village in Kongwa District. Trachoma was graded as zero if the ocular signs did not meet WHO criteria for TF (trachomatous inflammation: follicular) or TI (trachomatous inflammation: intense). Grade one TF or TI met the WHO criteria; grade two for TF was if there were 10 or more follicles size >0.5 mm in the tarsal conjunctiva, and TI grade two was present if all the deep tarsal vessels were obscured by inflammation.

### Serology for assessment of Trachoma-specific antibodies

The initial selection, expression, purification, and methodology for IgG antibody detection by Luminex technology of the chlamydial recombinant proteins CT694 and pgp3 antigen are described elsewhere [2]. Briefly, serum was eluted from dried blood spots and then incubated with chemically-modified microspheres (Luminex Corp., Austin, TX) conjugated to the trachoma antigens pgp3 and CT694. After washing out unbound serum antibodies, bound antibody was detected with biotinylated mouse anti-human IgG (clone H2; Southern Biotech, Birmingham, AL) and biotinylated mouse anti-human IgG_4_ (clone HP6025; Invitrogen, South San Francisco, CA), followed by *R*-phycoerythrin-labeled streptavidin (SAPE, Invitrogen, South San Francisco, CA). For detection of IgA antibodies in serum, total IgA was detected with 50 ng of biotinylated mouse anti-human total IgA (clone GA112, Zymed Life Technologies, Carlsbad, CA), followed by SAPE. Beads were suspended in 125 μl PBS, shaken, and immediately read on a BioPlex 200 instrument (Bio-Rad, Hercules, CA) equipped with Bio-Plex Manager 6.0 software (Bio-Rad).

### Determination of cut-offs for antibody positivity

Receiver operator curves (ROC) were generated for each antigen to determine cutoff values for IgA responses using finger prick sera, samples from 122 children from the United States, and dried blood spots from ten 1–9 year old Tanzanian children with ocular swabs that were PCR-positive for *C. trachomatis* DNA. The threshold for IgA positivity was determined to be a median fluorescence intensity minus background (MFI-BG) of 120 for pgp3 and 43 for CT694. The cutoffs for IgG responses were the same as previously described [2]; pgp3 had a cutoff value of 1024 and CT694 had a cutoff of 232.

### PCR

Eye swabs were collected for PCR analyses of *C. trachomatis* from all children at the baseline collection, with careful attention to avoid field contamination. Swabs were sent to the International Chlamydia Research Laboratory at Johns Hopkins University and tested for the presence of chlamydial DNA using Amplicor CT/NG (Roche, Basel, Switzerland). Details of laboratory processing were described elsewhere [6]. According to the manufacturer’s directions, the Amplicor test was positive if the optical density read at 450 nM was ≥0.8 and negative if the signal was <0.2 and equivocal if in-between. All equivocal tests were re-tested in duplicate, and only graded positive if at least one test was positive.

### Statistical analysis

Plots and descriptive analyses were conducted using GraphPad Prism 5.03 (GraphPad Software Inc., La Jolla, CA). Specificity and sensitivity were calculated for both antigens by receiver operator characteristic analysis. Comparisons of serological responses, clinical trachoma, PCR results for infection, and age groups were calculated with confidence intervals (CI) of 95% using Mann–Whitney tests and Wilcoxon matched pairs signed rank tests to generate p-values.

To assess the difference in mean percentage decreases of pgp3 and CT694 antigens following drug treatment, a linear regression model was developed in R version 3.0.1 (R Foundation for Statistical Computing, Vienna, Austria). Since the correlations between baseline and 6 month pgp3 and CT694 scores were greater than 0.8, we analyzed the response variable as a percent decrease within the 6 month time period which is acceptable based on previous research [10]. Our final models retained the age of the participant in order to calculate age-specific percent decreases and Schwarz’s Bayesian information criterion indicated gender did not improve model fit [11]. Hence, our final models include age as the only predictor. Results are presented as the mean percentage decrease of pgp3 and CT694 values broken down by age. The two-sided tests and confidence intervals are based on the 5% level of significance.

## Results

### Comparison of antibody responses, clinical pathology, and PCR detection of bacterial DNA in the conjunctiva at baseline

**Figure 1:**
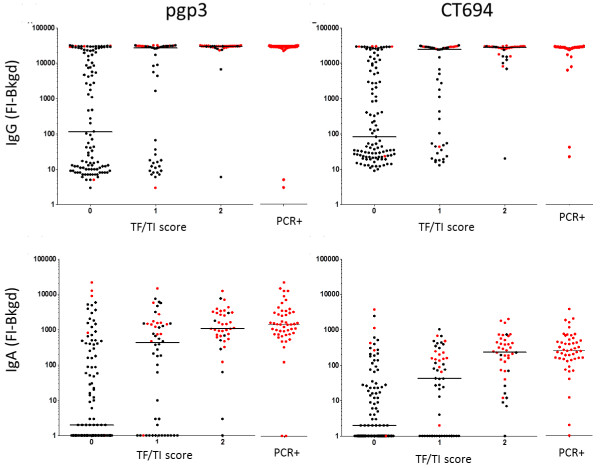
**Antibody response by clinical diagnosis and PCR.** Median responses are shown in median fluorescence intensity minus background (MFI-BG) for antibody response to pgp3 (left) and CT694 (right) in relation to clinical diagnosis. Antibody response to pgp3 and CT694 is shown by PCR positivity to the right of each graph. Responses shown in red indicate PCR positivity. IgG responses (top panels) and IgA responses (bottom panel) are shown. For IgG, N = 208 and for IgA, N = 184.

**Figure 2:**
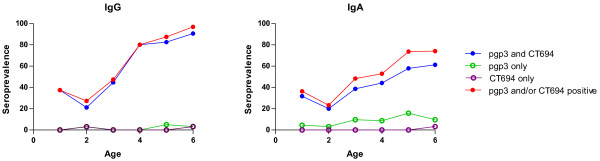
**Age prevalence curve of IgG and IgA responses at baseline.** DBS were collected from participants and analyzed for IgG and IgA levels by Luminex multiplex assay. Seroprevalence (% antibody positive/total) was plotted against age. Blue closed circles show% positive to both pgp3 and CT694 antigens; green open circles show% positive to pgp3 but not CT694; purple open circles show% positive to CT694 but not pgp3; and red closed circles show% positive to any antigen.

Of DBS collected from 264 total participants, 208 samples were tested for IgG antibodies at baseline. The remainder were excluded if samples were collected at only a single time point from a given individual or if that individual was <1 year of age, since antibody responses in this age range may reflect transfer of maternal IgG. From the subset of 208 samples, clinical prevalence of TF/TI was 47%, infection prevalence by PCR was 25%, and IgG seroprevalence was 64%. In accordance with previous studies, serology showed high sensitivity, as 49/51 (96%) of PCR-positive individuals tested positive for IgG antibodies against pgp3 and CT694 (Figure 1). By age 4, IgG seroprevalence was 81% and by age 6, 97% of participants had IgG responses against CT694 or pgp3 (Figure 2). The concordance between pgp3 and CT694 IgG responses was 95.4%(OR = 696.83; 95% CI = 149.02-6691 [12]).

Of the 184 samples that were tested for IgA antibodies, 99 (53.2%) had responses to pgp3, CT694, or both (Figure 2). Similarly to IgG, an age-specific increase in IgA responses was also observed (Figure 2). The peak IgA response was 74.2% among 6 year olds. The concordance between pgp3 and CT694 IgA responses was 81.8% (OR = 167.15; 95% CI = 39.10-1591.26 [12]). The majority of IgA-positive samples had responses to pgp3 (98/99) whereas fewer had responses to CT694 (82/99). Only one CT694 IgA + sample did not have a pgp3 IgA response. While 48/50 PCR-positive samples tested positive for IgA antibody against pgp3, only 46/50 PCR-positive samples (92%) were positive for CT694 IgA antibodies. The median IgA antibody response for children with normal ocular findings (a TF/TI score of zero) was significantly lower than those with observed ocular disease (a TF/TI score of two) for both pgp3 and CT694 (p < 0.001 for each antigen, Figure 1).

Comparison of IgG and IgA responses on the 184 samples for which both responses were measured showed that of those samples with IgG responses to either antigen (n = 122, 66%) , 21 (17%) were seronegative to both antigens for IgA, 23 (19%) were seronegative for pgp3 IgA, and 40 (33%) were seronegative for CT694 IgA. All IgA-positive samples had positive IgG responses; one IgA positive sample was positive for only IgG pgp3, while the rest were IgG positive to both antigens (data not shown).

No gender association in IgA or IgG response was observed (data not shown).

#### Kinetics of IgG and IgA responses after drug treatment

**Figure 3:**
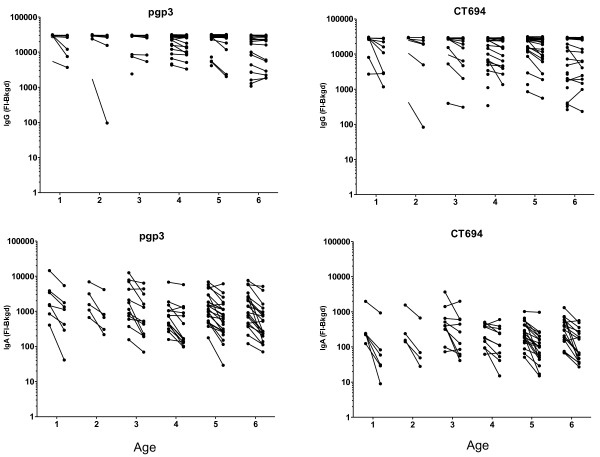
**Difference in individual IgG and IgA responses by age 6 months post-drug treatment.** DBS were taken prior to drug treatment with azithromycin and 6 months afterwards. IgG and IgA levels were measured by Luminex multiplex assay and data from paired samples were plotted using GraphPad Prism. Lines connect paired samples from the same individual.

Antibody responses in individuals at baseline and six months were compared in all individuals with a positive antibody response at baseline. IgG and IgA responses decreased for both pgp3 and CT694 when comparing paired baseline and six month post-treatment responses stratified by age (Figure 3). All age groups showed significant decreases in antibody responses from baseline to six months post treatment for both antigens, with the exception of 2 year old children, and 3 year old children for CT694 (Table 1).

**Table 1:** Decrease in antibody level 6 months after drug treatment stratified by age

**Age (years)**	**N**	**% pgp3 Ab + after 6 mos (CI)**	**P-value**	**N**	**% CT694 Ab + after 6 mos (CI)**	**P-value**
		**IgG**	**0.06**			**0.28**
**1**	8	22.24 (4.24, 40.25)	0.01	8	36.89 (10.46, 63.32)	0.002
**2**	5	9.64 (−13.14, 32.41)	0.83	6	32.25 (1.73, 62.77)	0.03
**3**	14	4.94 (−8.67, 18.54)	0.91	15	18.36 (−0.94, 37.67)	0.07
**4**	22	9.07 (−1.79, 19.93)	0.15	23	15.59 (0.00, 31.18)	0.05
**5**	30	12.01 (2.72, 21.31)	0.005	28	16.40 (2.27, 30.53)	0.01
**6**	26	4.01 (−5.98, 14.00)	0.86	25	0.60 (−14.36, 15.55)	1.00
		**IgA**	**0.003**			**0.0006**
**1**	7	60.08 (31.36, 88.80)	<0.0001	6	75.06 (40.00, 100.00)	<0.0001
**2**	5	60.11 (26.12, 94.10)	<0.0001	4	68.85 (25.90, 100.00)	0.0003
**3**	12	47.91 (25.97, 69.85)	<0.0001	10	34.25 (7.08, 61.41)	0.01
**4**	14	41.91 (21.60, 62.22)	<0.0001	11	30.93 (5.03, 56.83)	0.01
**5**	23	45.52 (29.67, 61.37)	<0.0001	20	53.66 (34.45, 72.87)	<0.0001
**6**	20	52.53 (35.53, 69.52)	<0.0001	17	53.01 (32.17, 73.84)	<0.0001

Values show the mean percentage drop in antibody median fluorescence intensity-background (MFI-BG) for IgG (top section) and IgA (bottom) from baseline to 6 months after drug treatment in paired samples. 95% confident intervals are shown in parentheses. P values testing for a difference from zero (no change) of < 0.05 were considered significant. P values in bold test for an average difference from zero across all age groups.

At six months post-treatment, IgA responses decreased across all ages for both pgp3 and CT694 (Figure 3). While responses decreased, no children reverted to seronegative status. The mean decline in antibody for antibody-positive children decreased with age. The greatest decline for pgp3 IgA responses was seen in the one-year-old children, while the mean decline was smaller in the six-year-old children (decline = 325.2 (60.08%) for one-year-old children, and 145.9 (52.53%) for six-year-old children). A similar trend in the decline of the antibody response was seen in CT694 among antibody positive individuals (decline = 35.85 (75.06%) for one-year-old children and change = 31.34 (53.01%) for six-year-old children).

## Discussion

Seroprevalence has the potential to be useful for decision-making at multiple stages of trachoma control programs, from mapping to stopping MDA to long-term monitoring and evaluation. We are focusing on serological testing in terms of making decisions to cease MDA and long-term surveillance, since recent funding from the Department for International Development (DFID) in the United Kingdom to map over 1200 districts in over 30 countries suspected to be trachoma-endemic (http://www.sightsavers.org) makes the validation of serological tools for mapping a low priority. The program end-point is not the interruption of trachoma transmission, but the elimination of blinding trachoma as a public health problem [13]. Recrudescence of the disease long-term is still possible given the right conditions; therefore long-term monitoring strategies that can be easily integrated into ongoing national surveillance systems for disease and/or vaccine coverage offer the best option for surveillance once the currently defined end-points have been met and elimination programs have ceased.

In previous studies [2], IgG responses to *C. trachomatis* antigens were measured in children following multiple rounds of MDA. IgG responses correlated with ocular pathology and infection status, but also increased with age, making it difficult to determine if serological responses were due to an active or previous infection or to assess the impact of multiple rounds of MDA. The study reported here provides a first look at antibody responses in a high prevalence community before MDA. Baseline analysis showed good correlation between IgG responses, increasing TF/TI score, and PCR-positive specimens. In areas of high TF prevalence, IgG serology shows that well over 1/3 of children age 3 and under have been exposed to trachoma antigens, while almost 100% of children age 6 have been exposed. Analysis of IgG responses 6 months after MDA showed a significant decrease in antibody levels (represented here by MFI) in all age groups, but no instances of seroreversion, within 6 months following treatment. The lack of a significant decline in IgG responses is likely due to the presence of long-lived memory B cells or plasma cells induced by multiple exposures to ocular chlamydia experienced by children in endemic regions [14]. One could hypothesize from the data that the steeper decline in IgG responses after treatment in younger children is related to a lower overall antigen load experienced as a result of fewer exposures, and that an increase in the number of exposures over time repeatedly boosts memory B cells and plasma cells to become long-lived. Data from murine models [15,16], vaccination for smallpox [17-19] and other viral infections [20], and examination of humoral responses from 1918 influenza pandemic survivors nine decades later [21] have all shown that plasma cells can be incredibly long-lived, being present decades after the initial stimuli. However, it is possible that the local rather than systemic nature of ocular chlamydia infection requires multiple exposures to generate long-lived plasma cells to maintain high levels of circulating antibody.

IgA responses were also examined to determine if mucosal responses may be a better indicator of recent infection, as reported for *Campylobacter* infection [22,23]. Fewer children in this study had IgA responses than IgG responses, although this difference was more pronounced in older age groups. Virtually the same percentage of 1–3 year olds had IgG and IgA responses (39.4 vs. 36.1%, respectively), while fewer 4–6 year olds had IgA responses (86.4% IgG + compared to 64.1% IgA+). The MFI for positive IgA samples was overall about 1–1.5 log fold lower than for positive IgG responses, which is to be expected based on the overall lower IgA titers in serum than IgG [24,25]. IgA responses are related to the total antigen dose, whether a large bolus or repeated infections [26], so the lower percentage of children seroprevalent for IgA may represent a lower antigen load and failure to seroconvert to IgA positivity. Following drug treatment, IgA levels decreased at each age group more so than the corresponding IgG responses. In children between the ages of two and five, the decrease in IgA responses to CT694 were not as significant as other age groups, and low samples sizes may have contributed to the variance in slopes for these groups. The large variance in responses, in conjunction with a low sample size, does not produce an ideal normal distribution to compare the mean antibody responses from baseline to six months post treatment (data of remaining slopes not shown). While IgA responses can be long-lived, IgA levels will decline following exposure to other mucosal organisms [26], which may also contribute to the low IgA titers, seroprevalence, and more significant post-treatment decline than IgG.

The use of IgA analysis in a programmatic context poses some problems. IgA is the most prevalent isotype in mucosal surfaces but is a minor antibody isotype in the blood, which will be the sample type almost certainly used for large scale monitoring and evaluation of trachoma control programs. From a technical standpoint, testing for IgA poses additional problems for high through-put surveillance of large populations. IgG depletions prior to IgA analysis is recommended due to the lower serum concentrations of IgA than IgG [24,25] and this additional step adds significantly to time and costs. Testing for IgA also negates the ability to multiplex with IgG responses of other disease antigens. IgM may be a better option for picking up recent infections, but the same obstacles to using this in a high-throughput, integrated platform as IgA remain. From a technical standpoint, IgA assays in this study had higher CVs than IgG assays did, with almost a quarter of the samples having CVs of greater than 15% for one or both antigen(s). This is likely due to the low concentration of IgA in the serum [26] and possibly through the binding of the more abundant IgG to antigen, sterically hindering the binding of IgA in the assay. This unexpected technical difficulty represents an additional obstacle to using IgA serology on a broad platform for monitoring and evaluating trachoma programs.

## Conclusions

The lack of seroreversion 6 months after drug treatment shown in this study confirms that surveillance will need to focus on very young children born after MDA cessation. It is possible that 6 months is too short a time frame to detect seroreversion; that this may occur after interruption of transmission and studies are underway to address this. Even in the absence of seroreversion post-treatment, the absence of antibody responses among young children will be a strong indicator of program impact. Despite the large differences in the odds ratios, the overlapping CIs indicate no significant differences in the agreement between the IgG and IgA responses to the two antigens. A larger sample size will provide definitive information to determine if looking at only concordant responses in the assay will provide the best estimate of transmission. Data provided herein and in ongoing studies will help inform the best strategy for employing trachoma serology for post-MDA surveillance.

## Abbreviations

Ig: Immunoglobulin; DBS: Dried blood spot; OR: Odds ratio; CI: Confidence interval.

## Competing interests

The authors have no competing interests to declare.

## Authors’ contributions

EBG and SMGM performed multiplex assays and analyzed data; AJS and RW performed statistical analysis; LD performed PCR; HM organized field data collection; BM, CG, SKW, PJL, and DLM conceived and designed the study; DLM wrote the paper. All authors read and approved the final manuscript.

## Pre-publication history

The pre-publication history for this paper can be accessed here:

http://www.biomedcentral.com/1471-2334/14/216/prepub
